# Native American Elders’ Experiences during the COVID-19 Pandemic: Case Studies

**DOI:** 10.1093/geroni/igab046.3214

**Published:** 2021-12-17

**Authors:** Bo Xie, Kristina Shiroma, John Lowe, Atami De Main, Nathan Davis

**Affiliations:** 1 The University of Texas at Austin, Austin, Texas, United States; 2 The University of Texas at Austin, The University of Texas at Austin, Texas, United States; 3 The University of Texas at Austin, Menard, Texas, United States

## Abstract

The COVID-19 pandemic has affected community-dwelling elder adults’ experiences during the COVID-19 pandemic. We report 4 case studies of Native American elders’ pandemic experience. Participants were recruited from community-dwelling older adults in Central Texas. Data collection took place via in-depth, semi-structured telephone interviews during June-August 2020. Four of the participants self-identified as Native American. Three of them were male; between the ages of 74 and 75; had at least some college education. The fourth Native American elder was a 68 year-old female with some college education. All four participants were coping well with everyday life during the pandemic. Connectedness emerged as the overarching theme among the 4 cases. Regular communication with their families was expressed as most important. A variety of communication technology was used to maintain contact with family members such as phone calls, texting, email, and video chat services particularly Zoom, FaceTime, and Facebook Video Chat. Challenges with using these technologies were also frequently reported. The participants expressed they did not feel a sense of increased loneliness or loss of being connected. Another theme emerged related to surviving the impact of the pandemic. Having the vaccine accessible along with financial resources necessary to sustain essential needs were most frequently expressed by the participants. These findings have implications for community interventions and policies that support the provision of mechanisms for Native American elders to maintain a sense of connectedness, including the adoption and use of communication technology, during times of crises such as pandemics and natural disasters.

